# Comparison between bracing and hollowing trunk exercise with a focus on the change in T2 values obtained by magnetic resonance imaging

**DOI:** 10.1371/journal.pone.0240213

**Published:** 2020-10-08

**Authors:** Yuki Muramoto, Hironobu Kuruma

**Affiliations:** 1 Department of Rehabilitation, Katsushika Edogawa Hospital, Katsushika, Japan; 2 Department of Physical Therapy Science, Tokyo Metropolitan University Graduate School of Human Health Sciences, Tokyo, Japan; Ritsumeikan University, JAPAN

## Abstract

The purpose of this study was to compare the muscle activity of Bracing and Hollowing trunk exercises by means of T2 values using MRI. Subjects were 19 healthy adult males, of whom 10 (with mean height ± SD: 172.3 ± 4.7 cm, mean weight ± SD: 64.3 ± 5.4 kg, mean age ± SD 21.5 ± 1.9 years) performed hollowing and 9 (with mean height ± SD: 171.3 ± 2.1 cm, mean weight ± SD: 68.5 ± 11.7 kg, mean age ± SD: 23.0 ± 2.6 years) performed bracing. They were assessed using MRI. The imaging was completed using Osirix software, which measured T2 values from the transversus abdominis (TrA), internal oblique (IO), external oblique (EO), and multifidus (MF) muscles. Subsequently, T2 values recorded before the exercise were compared with those recorded after the exercise to evaluate the extent of change effected by exercise on the muscles. MRI T2 values indicated that the TrA and IO regions were activated to a significantly greater degree after bracing. No significant changes occurred in any muscle before and after hollowing. It was determined that the activity of the deeper trunk muscles was higher in bracing than in hollowing on comparing the T2 values obtained in the MRI.

## Introduction

The deep muscles of the trunk, such as the transversus abdominis (TrA), internal oblique (IO), external oblique (EO), and multifidus (MF) are involved in maintaining its stability in coordination with other superficiall muscles. TrA and IO attach to the spine through the thoracolumbar fascia, and contraction of the same increases the internal abdominal pressure and stability of the trunk [1]. Therefore, if muscles around the trunk do not function adequately, the lumbar region becomes unstable, and the risk of lower back pain increases [2]. An important way to increase trunk stability is to activate the truck muscle group voluntarily [3, 4].

In clinical and sports settings, exercises that withdraw the abdomen (hollowing) and inflate the abdomen (bracing) are generally used to enhance trunk stability. Hollowing is particularly effective in activating TrA selectively [1]. The activity of the TrA is attenuated in those with chronic low back pain. This improves with hollowing, which increases the activity of this muscle. On the other hand, bracing is an exercise that works in collaboration with TrA, IO, and EO by inflating the abdomen [5]. Bracing is used to rehabilitate athletes because it increases abdominal pressure more than hollowing [6, 7]. However, it has also been reported that bracing causes a reduction in the ability to maintain balance [8] and increased compression of the intervertebral discs [3]. Therefore, you need caution when you teach patients with low back pain. To date, there are no reports that compare the effects produced by bracing and hollowing quantitatively.

A surface electromyogram is used as an indicator in the research intended to measure muscle activity in the trunk because it is suitable for evaluating muscle activity in superficial muscles [9]. However, it is not suitable for assessing the activities of deep muscles because it cannot distinguish that of other muscles from the activities of muscles like TrA. Otherwise, Indwelling EMG is specific to the target muscle, and the normalized intramuscular signal is representative of the entire muscles [10, 11]. However, this is an invasive method of measuring muscle activation, we cannot perform without medical doctor.

In recent years, changes in T2 values in MRI have been used as an index for evaluating the activity of deep muscles [12–14]. T2 values increased after exercise, even after more time on MRI, clearly reflecting the effect of exercise [15, 16]. The effect of the exercise were reported to last at least 20 minutes [17]. Another study clarified that T2 changes are greater than the baseline when they are measured 24 hours and 48 hours later [18]. T2 value can quantify all muscle activities within the imaging range by exploiting the process by which exercise induces signal changes that result primarily from increases in T2 relaxation time of water in the tissues [19, 20]. T2 changes measured with MRI have high reliability [21, 16]. Therefore, because the prolongation in T2 relaxation time could easily be used as a non-invasive, quantitative measurement for muscle activity of the deep muscles, this technique is an excellent tool for assessing the extent of muscle activation following the performance of a task.

The purpose of this study was to compare the muscle activity of bracing and hollowing trunk muscles by means of T2 values using MRI.

## Methods

### Subjects

Subjects were 19 healthy adults. The bracing group comprised of 10 participants (with mean height ± SD: 172.3 ± 4.7 cm, mean mass ± SD: 64.3 ± 5.4 kg, mean age ± SD: 21.5 ± 1.9 years), and the hollowing group comprised of 9 participants (with mean height ± SD: 171.3 ± 2.1 cm, mean mass ± SD: 68.5 ± 11.7 kg, mean age ± SD: 23.0 ± 2.6 years). These subjects do not have any exercise habits, nor did they receive instructions on trunk training. Before conducting the study, the subjects were randomly assigned to two groups. The subjects drew lots to determine which group they would belong. After we determined the task to be performed, we asked them to practice it for 10 minutes under the guidance of the examiner to ensure that they perform it perfectly.

The researchers recruited participants by posting a notification on the bulletin board of the university. They enrolled volunteers who had not experienced lower back pain for the past six months. They should not have had any neurological symptoms in the lower limbs at the time of measurement, and should not have had surgery of the spinal column. Information about all experimental procedures was provided and informed consent was obtained in verbally before the study was conducted. The experimental procedure was approved by the Research Safety Ethics Review Committee of Tokyo Metropolitan University (approval number: 17064).

### Experimental procedure

Participants underwent a baseline MRI imaging that lasted for approximately 20 minutes initially. Then, they performed their experimental exercise that lasted for approximately 10 minutes in the reception area of the MRI suite. They went back into the MRI room immediately after they finished the exercise, and another scan was carried out for approximately 20 minutes.

### Exercise method

Participants’ abdomen was kept exposed to check their abdominal pressure, while the researcher placed his fingers on the subject’s sidewall [22]. Participants exercised for 50 s and rested for 10 s in one set. They completed 10 such sets.

### Tasks

The tasks adopted here were performed following the procedure used in a prior study [22]. Brief descriptions of the tasks are provided below.

#### Abdominal bracing

**Fig 1:**
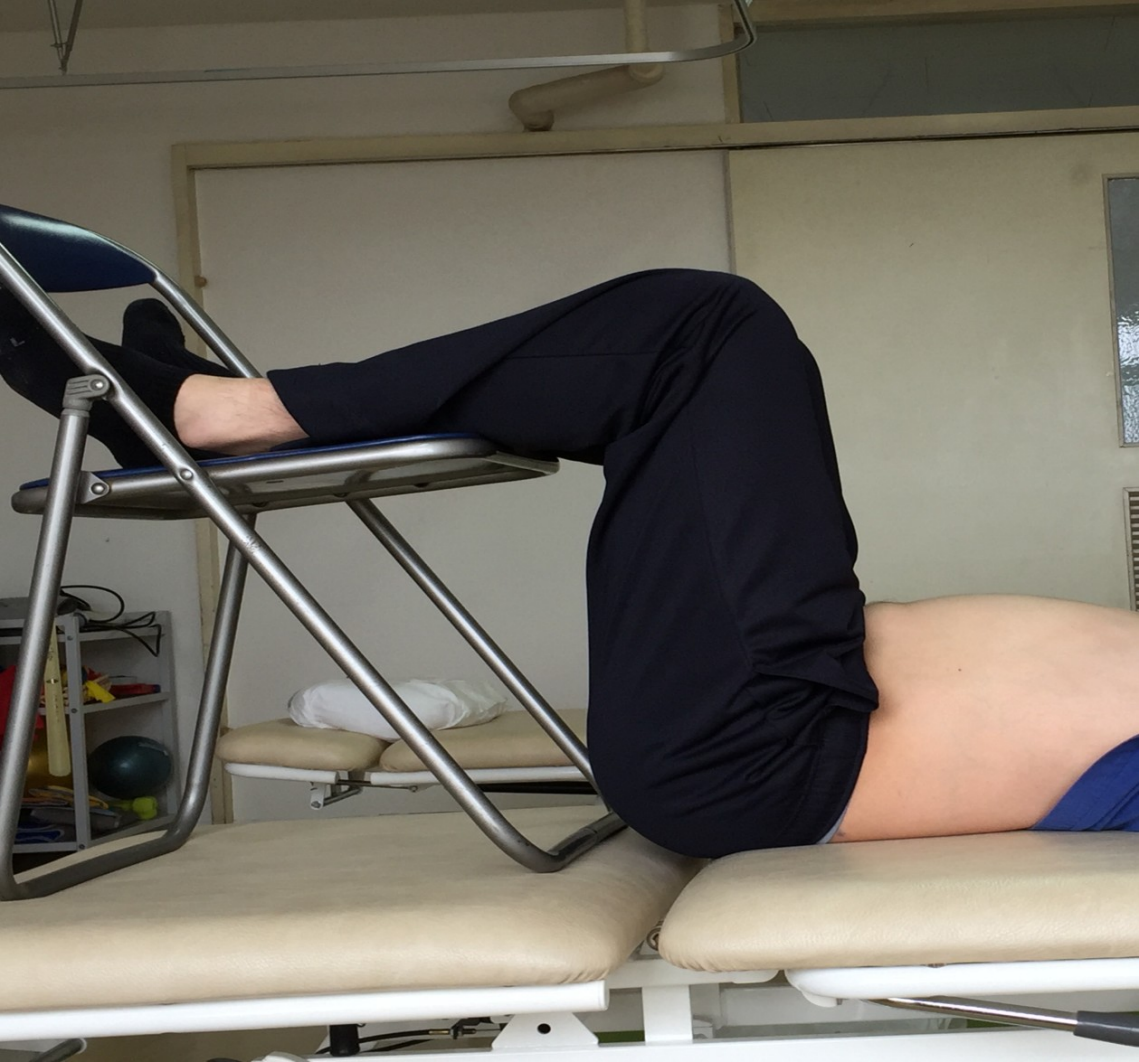
Bracing exercise. Participants were instructed to inflate abdomen as much as they can.

Subjects lay supine on the floor with their hip and knee placed at 90° flexion on the chair. They were instructed to expand the abdomen and breathe, as usual (Fig 1).

#### Abdominal hollowing

**Fig 2:**
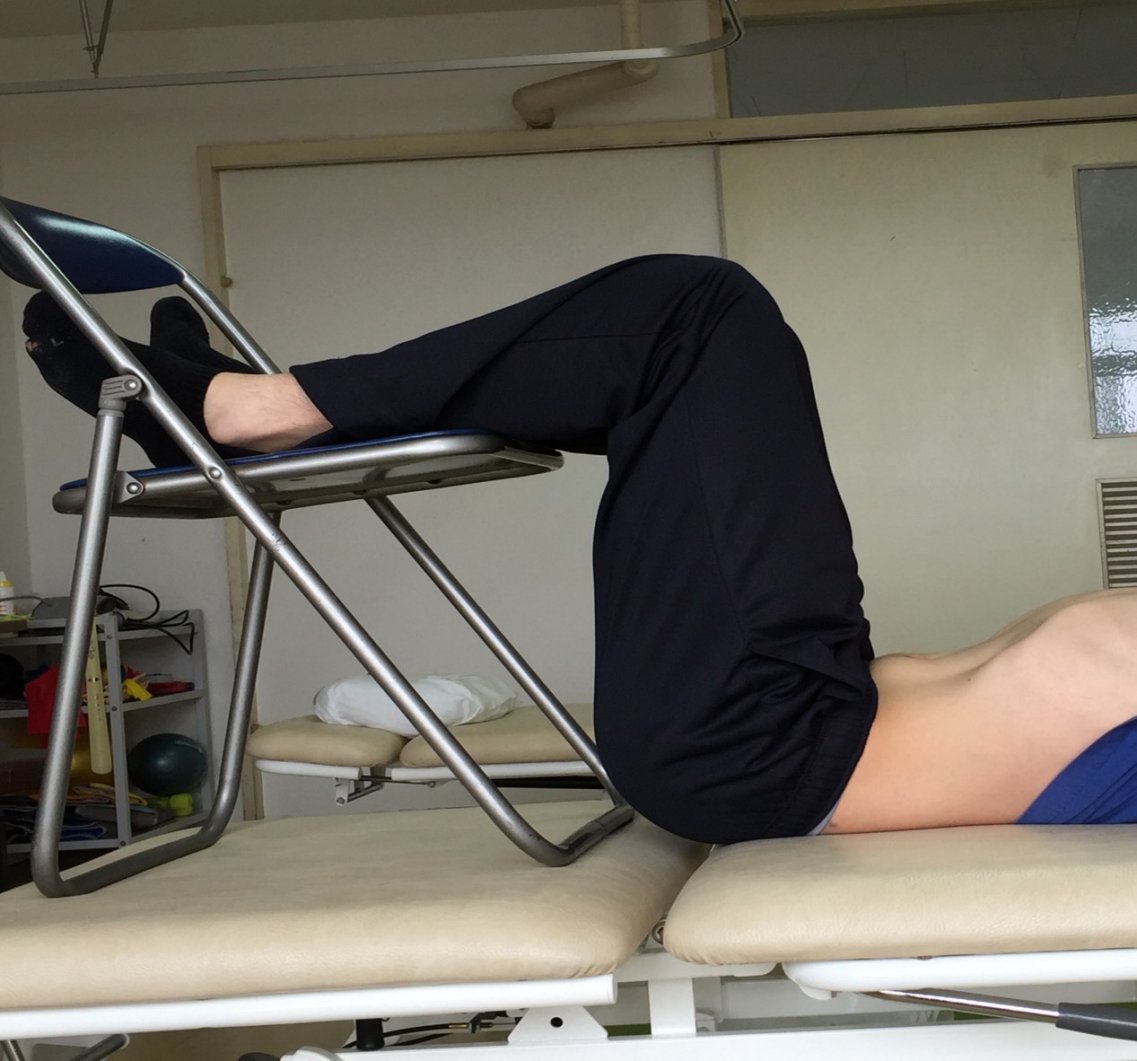
Hollowing exercise. Participants were instructed to push abdomen as much as they can.

Maintaining the same position as for the abdominal bracing, subjects were instructed to draw the lower abdomen into the spine and breathe, as usual (Fig 2).

### MRI imaging

Imaging was carried out at rest with the knee joint in mild flexion. Participants were advised to put on headphones to reduce the loud sounds generated from the MRI during the experiment. The abdomen at the level of lumbar spine 4 (L4) was imaged, and T2 values calculated. During MRI, participants were monitored clinically throughout the procedure, and experimenters were advised to stop the procedure if any abnormality occurred. In addition, participants were also allowed to stop the procedure by themselves via a hanging buzzer.

The MRI device was Achieva 3.0T (Philip Co., Ltd.), and one of the collaborators of this experiment performed imaging. We used SENSE TORSO coil (Philip Co., Ltd.) for MRI and adopted the following sequence parameters of MRI T2 values: 13–68 ms for TE, 3000 ms for TR, 90 degrees for flip angle, 340 mm for field view, 256 × 256 for matrix size, 5.0 mm for slice thickness, and 27 for the number of slices.

**Fig 3:**
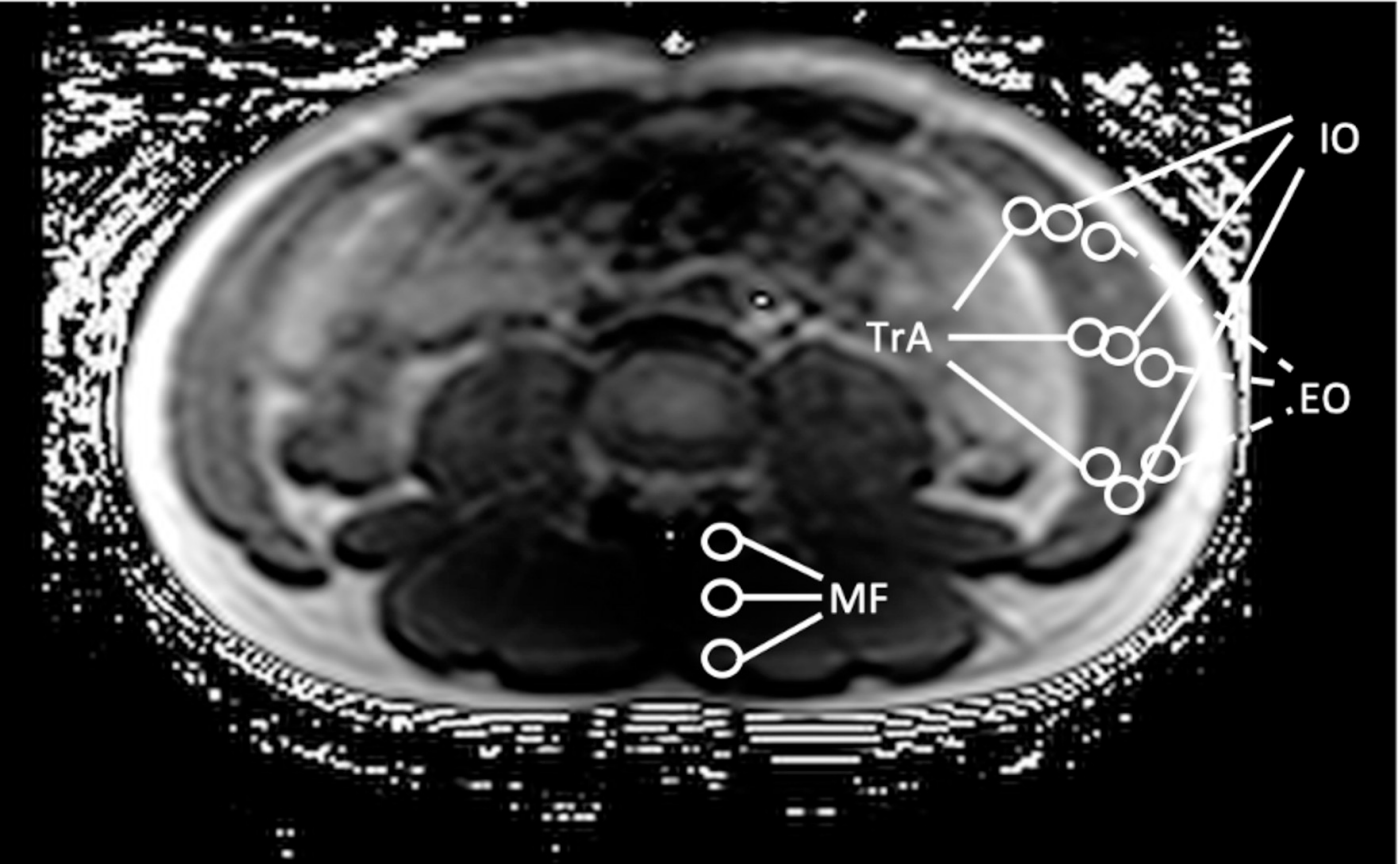
T2 measurements of trunk muscles on a T2 calculated map. TrA: Transverse abdominis, IO: Internal oblique, EO: External oblique, MF: Multifidus.

The MRI data were incorporated into Osirix (NEWTON GRAPHICS Co., Ltd.). T2 values of TrA, IO, EO, and multifidus (MF) were measured by taking an average of the three points in the ROI, and the workload for each muscle was expressed as a T2 value in milliseconds (Fig 3).

### Statistical analysis

MRI T2 values before and after the exercise were compared using two-way (time * group) ANOVA with repeated measures. Significant main effects were compared using the MANOVA post hoc test. We analyzed all data using SPSS (version 22.0, IBM Corporation, Japan) with a priori alpha level of .05.

## Results

Two-way (time * group) ANOVA with repeated measures showed a significant interaction for TrA and IO (Table 1) with a simple main effect test. The simple main effect of the bracing group was significant for the TrA (Mean difference = 3.84, 95%CI = 2.52–5.15, F = 25.27, partialη^2^ = 0.11, p< .01). The simple main effect of the bracing group was significant for the IO (Mean difference = 3.66, 95%CI = -2.43–4.90, F = 25.58, partialη^2^ = 0.12, p<. 01).

**Table 1:** Two-way repeated-measures analysis of variance for comparisons between time and group (mean±(standard deviation)).

	TrA		IO		EO		MF	
	Bracing (n = 10)	Hollowing (n = 9)	Bracing (n = 10)	Hollowing(n = 9)	Bracing (n = 10)	Hollowing(n = 9)	Bracing (n = 10)	Hollowing (n = 9)
Pre, ms	35.0	38.1	34.7	35.4	33.1	34.9	42.5	43.3
	(3.3)	(3.4)	(2.6)	(2.4)	(3.9)	(3.4)	(3.2)	(4.3)
Post, ms	38.8	39.1	38.3	36.3	33.2	35.3	44.6	43.6
	(3.6)	(4.4)	(3.9)	(4.5)	(3.7)	(3.9)	(3.2)	(4.3)
f	6.64		7.19		0.46		1.46	
p	0.01		0.01		0.5		0.23	

*p <0.05.

Note: TrA; transverse abdominis IO; internal oblique EO; external oblique MF; multifidus.

These results revealed that TrA and IO had increased T2 values on MRI after bracing.

## Discussion

The bracing group had a significant increase in T2 values of TrA and IO after exercise. Bracing is known to use EO, IO, and erector spinae more than hollowing [23]. The deep abdominal muscles contribute to the stability of the spinal column [24]. The T2 values of these muscles changed significantly after the exercise.

From the results of our study, it is concluded that hollowing did not produce any change in any of the assessed muscles after the exercise (Table 1). Hollowing was unable to adequately activate trunk muscles after a 10-minute exercise. Hollowing was reported to selectively activate the TrA [1]. It was reported that the change in the activation of TrA was 1.4 times higher than the rest in a study done to determine the muscle thickness during hollowing and rest, using ultrasound imaging. It was 1.1 times more in the IO and EO [25]. The TrA was significantly thicker during abdominal hollowing than without hollowing in four-point kneeling [26]. However, the T2 values of the TrA did not change immediately after exercise. Therefore, hollowing in a prone position is inadequate for deep trunk muscle exercise. It is reported that hollowing does not increase intra-abdominal pressure in the same way as bracing [9]. The Co-contraction of the TrA and IO is necessary to stabilize the trunk [27]. In hollowing, the trunk may not be stable because the deep trunk muscles are not active.

From the results of our study, the T2 values of the TrA and IO are significantly increased after the bracing (Table 1). It was reported that comparison before and after eccentric contraction training to the quadriceps muscle, the partial η^2^ was 0.14–0 17. The load of Bracing was probably comparable to that exerted by the eccentric contraction on the quadriceps muscle. Therefore, it seems likely that a 10-minute bracing exercise activates deep muscles in the trunk. It is reported that if both bracing and hollowing exercises were performed for six weeks, the thickness in the TrA, IO, and EO changed in the bracing group, whereas thickness in the TrA alone changed in the hollowing group [28]. Therefore, exercises performed by the bracing group, which included plank and side plank, were believed to have resulted in changes in the EO. MF was not evaluated. Another study compared intra-abdominal pressure and electromyogram of trunk muscles and found that the bracing group had higher intra-abdominal pressure than the hollowing group [9]. In addition, higher values were recorded not only for rectus abdominis in the electromyogram but also in the IO, EO, and MF in the bracing group [9].

Based on these reports, we can infer that bracing is more effective than hollowing for the activation of trunk muscles.

The results of our study agree with those of previous studies with respect to the bracing group. This group experienced a high degree of activation in the TrA and IO. A study compared the stability of the trunk when maintaining hip abduction while performing side plank between bracing and hollowing. The study reported that bracing increased muscle activity of IO and MF and reduced the rotation of the trunk [4]. McGill reported that bracing activated the trunk muscles contributing to trunk stability, the transversus abdominis contributing to spinal column stability [29]. However, it occurred via compression of the intervertebral discs [3]. The results of this study indicate that bracing is more effective in stabilizing the spinal column than hollowing because it affects the TrA, IO. Therefore, bracing may be more effective than hollowing in rehabilitating athletes.

There are several limitations to this study. First, the subjects were randomly divided into groups, but there was a difference in resting T2 values due to the variability of the subjects. Second, MRI T2 values change with higher muscle fatigue relative to the exercise [18, 30], and because hollowing is a low-intensity exercise that selectively uses TrA [1], the results of the current study may not have reflected the change. Therefore, it may be necessary to increase the duration of the exercise or to exercise until the subject feels fatigued.

In conclusion, the study compared bracing and hollowing using T2 values in MRI. Bracing was more effective than hollowing in increasing the activity of deep muscles of the trunk. Thus, bracing is suitable for athletes who require trunk stability. Knowledge regarding which muscle is used during bracing and hollowing can help clinicians prescribe exercises tailored to each individual. Ultimately, this may lead to better treatment of back pain and higher performance rates in athletes.

## Supporting information

S1 File (XLSX)
