# Correction: Comparison between bracing and hollowing trunk exercise with a focus on the change in T2 values obtained by magnetic resonance imaging

**DOI:** 10.1371/journal.pone.0267928

**Published:** 2022-04-27

**Authors:** Yuki Muramoto, Hironobu Kuruma

There is an error in reference 5. The correct reference is: Masashi K, Norihiro S, Hidetsugu N.Regular change in spontaneous preparative behaviour on intra-abdominal pressure and breathing during dynamic lifting. Eur J Pppl Physiol. 2014; 114(11):2233–9
